# Advances in the Multimodal Management of Pediatric Arteriovenous Malformations: A 10-Year Review

**DOI:** 10.3390/life15101620

**Published:** 2025-10-17

**Authors:** Ammar Saloum, Yusor Al-Nuaimy, Denise Baloi, Michael Karsy, Mehrdad Pahlevani, Brandon Lucke-Wold

**Affiliations:** 1 College of Human Medicine, Michigan State University, Lansing, MI 48824, USA; saloumam@msu.edu (A.S.); baloiden@msu.edu (D.B.); 2 College of Medicine, Ajman University, Ajman P.O. Box 255, United Arab Emirates; 3 Department of Neurosurgery, University of Michigan, Ann Arbor, MI 48109, USA; mkarsy@med.umich.edu; 4 Department of Neurosurgery, Jamaica Hospital Medical Center, New York, NY 11418, USA; 5 Department of Neurosurgery, University of Florida, Gainesville, FL 32608, USA

**Keywords:** Pediatric arteriovenous malformations, microsurgery, stereotactic radiosurgery, embolization, multimodal management, recurrence, rupture risk, neurosurgery

## Abstract

Pediatric brain arteriovenous malformations (AVMs) are rare but high-risk vascular anomalies associated with substantial morbidity and mortality due to their elevated lifetime risk of rupture. Over the past decade, advances in microsurgical resection, stereotactic radiosurgery (SRS), and endovascular embolization have reshaped the management landscape, yet treatment remains highly individualized and controversial, especially in unruptured cases. This narrative review synthesizes findings from 20 eligible studies published between 2015 and 2025, examining outcomes across different modalities. Microsurgical resection demonstrated the highest immediate obliteration rates (>95%) in low-grade, accessible, ruptured lesions, but recurrence rates remain disproportionately higher in children (up to 29%). SRS achieved obliteration rates of 63–72% in Spetzler–Martin (SM) I–III lesions with low complication and recurrence rates, although outcomes were less favorable for higher-grade AVMs. Embolization alone provided limited curative potential but served as an important adjunct in multimodal therapy. Importantly, embolization prior to radiosurgery was associated with reduced obliteration rates and higher complication risks. Across modalities, hemorrhagic presentation often predicted better treatment response, while recurrence and long-term surveillance emerged as central challenges in pediatric care. These findings highlight the necessity of multidisciplinary, individualized management strategies and emphasize the importance of lifelong follow-up to mitigate recurrence risk and optimize outcomes.

## 1. Introduction

Pediatric arteriovenous malformations (AVMs) are rare congenital vascular anomalies characterized by direct arterial-to-venous shunting without an intervening capillary bed. Compared to adults, pediatric patients have a higher lifetime risk of rupture, leading to substantial morbidity and mortality [1,2]. Morphologic features such as periventricular location and smaller nidus size further increase hemorrhagic risk in children [3]. While AVMs in adults have been extensively investigated, the pediatric subset presents unique challenges due to developmental vascular dynamics, diverse clinical presentations, and the scarcity of large-scale, age-specific data [4].

Over the past decade, advances in neuroimaging, endovascular techniques, and microsurgical strategies have significantly improved outcomes for these lesions [1,5,6]. However, treatment selection remains highly individualized, frequently relying on extrapolation from adult data or small pediatric series. The absence of universally accepted guidelines underscores the need for a consolidated review of contemporary literature. In addition, emerging work has begun to integrate molecular insights and precision-medicine approaches into the understanding of pediatric AVM [7]. This review synthesizes evidence from the past ten years, focusing on treatment outcomes for pediatric AVMs across different modalities.

## 2. Materials and Methods

This review was conducted as a narrative review of pediatric intracranial arteriovenous malformations (AVMs). A comprehensive search of PubMed, Scopus, and Embase was performed for studies published between 1 January 2015 and 1 July 2025, using combinations of keywords such as “pediatric brain arteriovenous malformations,” “arteriovenous fistulas,” “microsurgery,” “radiosurgery,” and “embolization.” The search was supplemented by manual screening of reference lists from retrieved articles to identify additional eligible studies.

Studies were eligible if they included patients aged ≤18 years and reported treatment outcomes. Exclusion criteria were case reports or case series with fewer than three patients, animal studies, conference abstracts, and studies without outcome or follow-up data.

Screening was performed independently by three reviewers (AS, DB, YN), with disagreements resolved by consensus. Data extracted included study design, sample size, treatment modality, outcomes, complications, recurrence, and follow-up duration.

## 3. Results

A total of 61 records were identified across PubMed, Scopus, and Embase, of which 30 studies published between 2015 and 2025 met the inclusion criteria. To avoid redundancy, smaller case series (<20 patients) are summarized narratively, whereas the largest and most representative institutional series, multicenter registries, and systematic reviews/meta-analyses are highlighted in Table 1.

### 3.1. Microsurgical Resection

Microsurgical resection was consistently reported as the most effective treatment for low-grade, surgically accessible AVMs. In the largest pediatric series to date, Eliava et al. analyzed 376 children, of whom 135 underwent surgery, and reported complete angiographic obliteration in 97.8% of cases, with no perioperative mortality and 89.6% of patients achieving favorable functional outcomes (GOS IV–V) [8]. Other institutional series confirmed high obliteration rates, typically between 89% and 100%, with perioperative morbidity of approximately 5% and minimal mortality when procedures were performed in high-volume centers [19].

Despite these favorable immediate outcomes, recurrence was a notable finding. Copelan et al. examined 115 patients under the age of 25 and observed a 21.4% recurrence rate at 5 years, with rupture at presentation strongly associated with recurrence [9]. Oulasvirta et al. documented sporadic recurrence in approximately 5% of patients, including one case in a child with hereditary hemorrhagic telangiectasia [20]. A systematic review by Järvelin et al. reported a recurrence rate of 29.4% in pediatric patients, compared with only 4.4% in adults [21]. Similarly, in a single-center series of 52 children, Shtaya et al. reported 96.6% angiographic obliteration following surgery, with favorable outcomes improving from 38.3% pre-treatment to 89.4% after intervention. Their accompanying literature review further demonstrated that approximately 82% of pediatric AVM patients present with hemorrhage, with a notable peak around puberty [10].

These findings collectively highlight microsurgery as the most reliable curative option, while underscoring the disproportionate recurrence risk in children compared with adults.

### 3.2. Stereotactic Radiosurgery

Stereotactic radiosurgery (SRS) has been reported in more than 2000 pediatric patients across systematic reviews and institutional series. Florez-Perdomo et al. conducted a meta-analysis and reported a pooled obliteration rate of 71.6%, with post-SRS hemorrhage in 2.46% of cases, neurological complications in 2.6%, and mortality below 1% [12]. In a focused series using CyberKnife(Elekta AB, headquartered in Stockholm, Sweden), Kim et al. evaluated approximately 80 children and documented a 79% obliteration rate for small-volume AVMs, though adverse radiation effects occurred in 31.6% of patients [14]. Outcomes for high-grade lesions were less favorable. Patibandla et al. reported a 10-year obliteration rate of only 35% in Spetzler–Martin IV–V AVMs, with an annual hemorrhage risk of 3.2% and permanent neurological deficits in 3.5% of cases [13]. Most recently, Thrash et al. performed a PRISMA systematic review and meta-analysis including 36 studies and over 3400 pediatric patients, reporting an overall obliteration rate of 63% at ≥1 year follow-up [15]. No significant differences were seen between Gamma Knife and LINAC platforms, while hemorrhagic presentation and prior procedures were associated with higher obliteration rates [22]. In parallel, the International Stereotactic Radiosurgery Society (ISRS) issued practice guidelines based on pooled pediatric data, confirming obliteration rates of ~65–75% with post-SRS hemorrhage in 2–3% of cases and reinforcing SRS as a safe and effective treatment for carefully selected pediatric AVMs [23]. Despite variability across series, once complete obliteration is achieved, recurrence after SRS remains exceedingly rare, with rates consistently below 1%; however, retreatment and staged radiosurgery, including typical indications (e.g., incomplete obliteration, persistent nidus in eloquent areas), is also advised [12].

### 3.3. Endovascular Embolization

Endovascular embolization has been applied in pediatric AVM management using different materials like MBCA, Onyx, detachable coils and other particles, however MBCA and Onyx are mainly used for partial or complete nidus obliteration while other particles and materials as well as the two former ones are used to treat accompanying vascular lesions depending on their locations, size and angioarchitecture of the accompanying vascular lesion which presents with short term or long term risk of rupture and bleeding like intra-nidal aneurysms and flow-related aneurysms adjacent to pediatric brain AVM [24]. In the Eliava series, 79 children underwent embolization, with angiographic cure achieved in 29.1% of cases and one procedure-related death, resulting in a mortality rate of 1.3% [8]. In a more recent single-center cohort of 26 patients, Borges de Almeida et al. reported complete angiographic exclusion in 42%, though most required subsequent microsurgery or radiosurgery for definitive cure. Recurrence occurred in 17%, and procedure-related complications were observed in 17%, yet all patients achieved good functional outcomes (mRS 0–2) [17]. Similarly, earlier institutional experiences documented cure rates of only 30–40%, with complication rates between 15% and 20%. The role of embolization prior to radiosurgery appears less favorable. In a multicenter registry of 539 children, Burke et al. compared outcomes of SRS alone with embolization followed by SRS and found a 10-year obliteration rate of 77.4% in the SRS-only group compared with 48.7% in those who had prior embolization, with significantly higher rates of radiation-induced changes in the latter group (16.7% vs. 9.0%) [16]. Experimental work further demonstrated that pre-SRS embolization may reduce radiosurgical efficacy by three plausible mechanisms: dose perturbation due to high-density embolic material (particularly Onyx), Nidus fragmentation, which can complicate target delineation, and obscured margins, leading to suboptimal SRS planning and incomplete nidus coverage [25].

### 3.4. Multimodality Approaches

Multimodal strategies are frequently required in pediatric AVM management, particularly for complex or high-grade lesions. In a UK cohort of 52 children, Aziz et al. reported that staged multimodal therapy (surgery, embolization, and/or radiosurgery) achieved an overall obliteration rate of 88% with no treatment-related mortality. Importantly, this study also incorporated quality-of-life measures, showing that ruptured presentation and infratentorial location were associated with worse psychosocial outcomes regardless of treatment modality [18].

Borges de Almeida et al. similarly observed that most pediatric patients treated with embolization ultimately required adjunctive surgery or radiosurgery to achieve complete obliteration, underscoring the reliance on multimodal sequencing rather than embolization alone [17].

In a multicenter registry of 539 children, Burke et al. compared outcomes of SRS alone versus embolization followed by SRS, finding a 10-year obliteration rate of 77.4% with SRS alone compared to 48.7% in the combination group. Prior embolization was also associated with higher rates of radiation-induced changes (16.7% vs. 9.0%), with experimental data suggesting that embolic agents such as Onyx perturb SRS dose distribution and may diminish treatment efficacy [16,25].

Finally, in a single-center series of 34 children with low-grade AVMs, outcomes were comparable between surgery alone and preoperative embolization followed by surgery. Complication rates did not differ significantly between groups, and long-term functional outcomes were favorable in both cohorts, suggesting that routine preoperative embolization may not provide additional benefit in low-grade lesions [26].

### 3.5. Recurrence

Recurrence is defined as the reappearance of arteriovenous shunting confirmed by digital subtraction angiography (DSA) after prior angiographic cure and emerges as a central challenge to pediatric AVM management. After microsurgical resection, recurrence rates ranged from 5% to 30%, particularly in patients presenting with rupture and in younger children [9,20,21]. In a 20-year single-institution cohort of 58 children, recurrence was observed in 12.8% of patients after apparent cure, with younger age (<7.5 years) identified as an independent risk factor and rupture at presentation appearing protective [27]. Hak et al. subsequently analyzed a prospective cohort of 70 children with ruptured AVMs and identified 10 recurrences, most frequently in cases where embolization was the definitive treatment. Infratentorial location and younger age were also significant predictors of earlier and more frequent recurrence. Their complementary systematic review and meta-analysis of 267 additional cases reported a pooled recurrence rate of 10.9%, reinforcing that embolization carries the highest recurrence risk, while recurrence following radiosurgical cure remains exceedingly rare [11]. Following confirmed obliteration by SRS, recurrence was rare, with rates consistently reported at less than 1% [23]. After embolization-only therapy, recurrence occurred in approximately 13% of cases, with a higher risk in small, ruptured AVMs [17]. Lifelong follow-up, particularly in pediatric patients, is advised due to documented cases of late recurrence.

## 4. Discussion

Pediatric brain arteriovenous malformations (AVMs) demand treatment strategies that balance immediate procedural safety with long-term durability. Compared with adults, children face a greater cumulative risk of hemorrhage due to longer life expectancy [1,3] and higher recurrence rates after treatment, likely reflecting vascular remodeling of the developing brain [9,10,20,21]. These differences limit the applicability of adult data, such as ARUBA, which supported conservative management in unruptured AVMs [28], and highlight the need for pediatric-specific frameworks. Recent series have also emphasized that early surgical intervention in ruptured pediatric AVMs can be performed safely and effectively in specialized centers, even in the acute phase of hemorrhage [29].

Microsurgical resection remains the most effective option for accessible low-grade ruptured AVMs, achieving the highest immediate cure rates [8,10]. However, recurrence rates in children are substantially higher than in adults, underscoring the importance of structured surveillance imaging. Hence, after microsurgical and embolization cases, we recommend DSA at 1–2 years post-treatment, followed by MRI/MRA every 3–5 years if no recurrence is detected [9,20,21]. Stereotactic radiosurgery (SRS) offers a minimally invasive alternative for deep or eloquent lesions, with favorable long-term outcomes once obliteration is achieved [12,13,14]. Its main limitation remains the latency to cure, which is typically 2–3 years and prolongs the risk of interval hemorrhage; therefore, surveillance should extend at least through the latency period (2–3 years) with serial MRI, and confirmation of obliteration by DSA is advised at or after year 3. Other limitations of SRS are adverse radiation effects (AREs) as symptomatic or radiologically evident brain tissue changes resulting from radiation exposure, including radiation-induced edema, necrosis, or neurological symptoms [15]. Endovascular embolization continues to play an important adjunctive role, particularly for targeting high-risk angioarchitecture or facilitating other therapies, but its standalone efficacy is limited, and pre-SRS embolization has been associated with worse outcomes [16,17,25].

The central challenge across modalities is recurrence. Even after angiographic cure, pediatric series document recurrence rates as high as 30% following surgery [9,20,21], compared with near-zero recurrence after SRS cure [23]. In a recent cohort and meta-analysis, Hak et al. reported a pooled recurrence rate of 10.9% in children, with infratentorial location, younger age, and embolization as definitive treatment identified as risk factors [11]. These findings collectively reinforce the need for lifelong imaging surveillance, with angiography within 1–2 years after treatment, followed by serial MRI/MRA at defined intervals [20].

Beyond individual modalities, multimodality strategies are increasingly recognized as essential in pediatric AVM management [30]. These approaches are detailed in Section 3.4, but collectively they highlight the importance of tailoring therapy to lesion complexity while balancing angiographic cure, functional outcomes, and quality of life.

**Figure 1:**
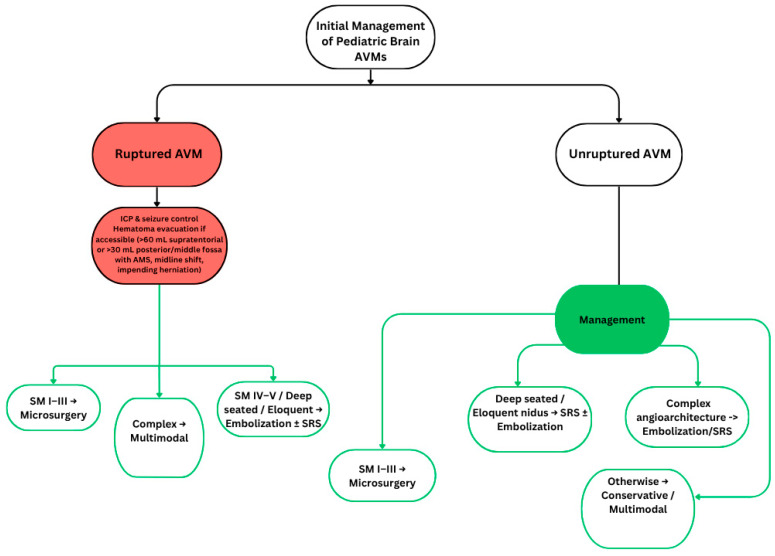
AVM Management Algorithm. SM = Spetzler–Martin grading system. Red boxes = Emergency management. Green boxes = Treatment/management options.

Optimal management must therefore be multidisciplinary and individualized. Decision-making should incorporate lesion grade, rupture status, and anatomical location, while family counseling should emphasize both treatment risks and the long-term commitment to follow-up [2,4,5]. Beyond individual treatment modalities, a practical synthesis of these findings is outlined in Figure 1. This proposed management algorithm integrates rupture status, Spetzler–Martin grade, and anatomical considerations to guide selection of microsurgery, radiosurgery, embolization, or multimodal approaches in pediatric AVMs.

### Limitations

This review is narrative in design and not meta-analytic, limiting quantitative comparisons. On the other hand, small sample sizes, the retrospective nature of the study, and potential selection bias are other key methodological limitations of our study. Included studies were heterogeneous in SM grades, rupture status, treatment modalities, and imaging endpoints, which has a constraint in drawing a uniform conclusion and comparability. Publication and language bias are possible. Pediatric-specific long-term neurocognitive and quality-of-life data remain underreported.

## 5. Conclusions

Management of pediatric AVMs requires a tailored, multimodal approach that accounts for lesion grade, rupture status, and anatomical accessibility [1,4,5]. Microsurgical resection remains first-line for surgically accessible ruptured low-grade lesions, offering the highest immediate cure rates [8,19]. Stereotactic radiosurgery provides a safe alternative for deep or eloquent AVMs, although patients must be counseled about the latency to obliteration and the ongoing risk of interval hemorrhage [12,13,14]. Endovascular embolization should be reserved for selected cases, primarily as an adjunct to other modalities, given its limited standalone efficacy and the potential negative impact of pre-SRS embolization on cure rates [16,17,25].

For unruptured AVMs, conservative management may be appropriate in carefully selected low-risk cases; however, the longer lifetime risk of hemorrhage in children demands individualized, multidisciplinary decision-making [6,7,28]. Lifelong surveillance is essential, as recurrence remains disproportionately high compared with adults [9,20,20,21]. A structured follow-up protocol—including angiography within 1–2 years post-treatment and serial MRI/MRA thereafter—should be standard.

Future efforts should prioritize refining risk prediction, establishing pediatric-specific treatment algorithms, and systematically evaluating long-term neurocognitive and quality-of-life outcomes. Ultimately, optimal outcomes will depend on close collaboration between neurosurgery, radiosurgery, and interventional neuroradiology teams, ensuring that treatment strategies are not only safe and effective but also durable over the long term [31].

## Figures and Tables

**Table 1:** Summary of Major Studies on Pediatric Brain AVM Management (2015–2025).

Author (Year)	Country/Setting	N (Patients)	Modality	Key Findings	Follow-Up
Eliava et al. (2020) [8]	Russia (single-center)	376	Microsurgery/Embolization	Microsurgical cure 97.8%; embolization cure ~30%; perioperative mortality 0%	5–10 yrs
Copelan et al. (2020) [9]	USA	115 (<25 yrs)	Surgery	Recurrence 21.4% at 5 yrs; rupture at presentation risk	5 yrs
Shtaya et al. (2017) [10]	UK (single-center)	52	Surgery	Cure 96.6%; favorable outcomes improved from 38.3% to 89.4%	3–5 yrs
Hak et al. (2022) [11]	USA (systematic review + cohort)	70 + 267	Multimodality/Recurrence	Pooled recurrence 10.9%; higher risk with embolization-only	Up to 10 yrs
Florez-Perdomo et al. (2024) [12]	Meta-analysis	2000+	SRS	Obliteration 71.6%; hemorrhage 2.5%; neuro deficit 2.6%; mortality <1%	≥3 yrs
Patibandla et al. (2017) [13]	USA	100+	SRS (SM IV–V)	Cure 35% at 10 yrs; hemorrhage risk 3.2%/yr	10 yrs
Kim et al. (2025) [14]	South Korea	~80	CyberKnife SRS	Obliteration 79%; 31.6% radiation effects	5 yrs
Thrash et al. (2025) [15]	Systematic review	3400+	SRS	Obliteration 63%; no GK vs. LINAC difference; hemorrhage cure	≥1 yr
Burke et al. (2021) [16]	Multicenter registry	539	Embolization + SRS	10 yr cure 77.4% (SRS only) vs. 48.7% (embolization + SRS); more radiation changes	10 yrs
Borges de Almeida et al. (2024) [17]	Portugal	26	Embolization	Cure 42%; recurrence 17%; complications 17%; all good mRS	3–5 yrs
Aziz et al. (2023) [18]	UK	52	Multimodality	Overall cure 88%; no mortality; QoL worse in ruptured/infratentorial cases	5–10 yrs

## Data Availability

No new data was created or analyzed in this study. Data sharing is not applicable to this article.

## Abbreviations

The following abbreviations are used in this manuscript:

Abbreviation	Meaning
AVM	Arteriovenous Malformation
SRS	Stereotactic Radiosurgery
GOS	Glasgow Outcome Scale
DSA	Digital Subtraction Angiography
MRI	Magnetic Resonance Imaging
SM	Spetzler–Martin (Grading System)
ARE	Adverse Radiation Effect
HFSRT	Hypofractionated Stereotactic Radiotherapy
mRS	Modified Rankin Scale
RIC	Radiation-Induced Complication
CTA	Computed Tomography Angiography
GKRS	Gamma Knife Radiosurgery
LINAC	Linear Accelerator
Onyx	Ethylene–vinyl alcohol copolymer embolic agent
ISRS	International Stereotactic Radiosurgery Society
